# Antibody effector analysis of prime versus prime-boost immunizations with a recombinant measles-vectored chikungunya virus vaccine

**DOI:** 10.1172/jci.insight.151095

**Published:** 2021-11-08

**Authors:** Roland Tschismarov, Raphaël M. Zellweger, Min Jie Koh, Yan Shan Leong, Jenny G. Low, Eng Eong Ooi, Christian W. Mandl, Katrin Ramsauer, Ruklanthi de Alwis

**Affiliations:** 1 Themis Bioscience GmbH, Vienna, Austria, a subsidiary of Merck & Co. Inc., Kenilworth, New Jersey, USA.; 2 Programme in Emerging Infectious Diseases, Duke-NUS Medical School, Singapore.; 3 Viral Research and Experimental Medicine Centre, SingHealth-Duke NUS (ViREMiCS), Singapore.; 4 Epidemiology, Public Health, & Impact, International Vaccine Institute, Seoul, Republic of Korea.; 5 Department of Infectious Diseases, Singapore General Hospital, Singapore.; 6 Vaccines and Viral Vectors, Lexington, Massachusetts, USA.

**Keywords:** Immunology, Vaccines, Adaptive immunity

## Abstract

Chikungunya is a mosquito-borne disease that causes periodic but explosive epidemics of acute disease throughout the tropical world. Vaccine development against chikungunya virus (CHIKV) has been hampered by an inability to conduct efficacy trials due to the unpredictability of CHIKV outbreaks. Therefore, immune correlates are being explored to gain inference into vaccine-induced protection. This study is an in-depth serological characterization of Fab- and Fc-mediated antibody responses in selected phase II clinical trial participants following immunization with the recombinant measles-vectored CHIKV vaccine, MV-CHIK. Antibody comparisons were conducted between participants who received prime and those who received prime-boost vaccine regimens. MV-CHIK vaccination elicited potent Fab-mediated antibody responses (such as CHIKV-specific IgG, neutralization, and avidity), including dominant IgG3 responses, which translated into strong antibody-dependent cellular cytotoxicity and antibody-dependent cellular phagocytosis. At 1 month, prime-boost immunization led to significantly greater responses in every measured Fab and Fc antibody parameter. Interestingly, prime-boost-elicited antibodies decreased rapidly over time, until at 6 months both vaccine regimens displayed similar antibody profiles. Nonetheless, antibody avidity and antibody-dependent cellular phagocytosis remained significantly greater following boost immunization. Our observations suggest that a prime-boost administration of MV-CHIK will be more appropriate for CHIKV-endemic regions, while a prime-only regimen may be sufficient for travel purposes or outbreak situations.

## Graphical abstract

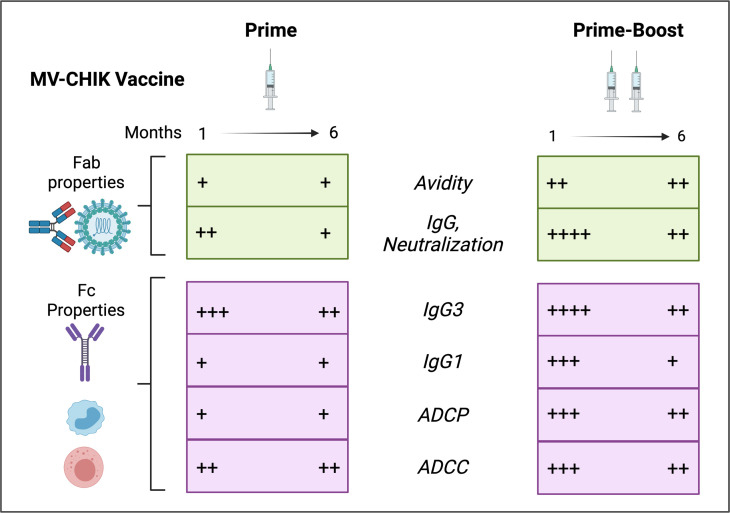

## Introduction

Chikungunya virus (CHIKV) is an alphavirus (positive-sense single-stranded RNA virus) that mostly causes an acute febrile illness referred to as chikungunya fever. While this disease is rarely lethal, severe polyarthralgia is a common symptom that can persist for months or even years in a substantial fraction of patients, causing long-term debilitating morbidity and imposing a massive burden on public health systems (1, 2). Since its original isolation in the 1950s, the virus has spread to over 100 countries and territories in Africa, Europe, Asia, and the Americas, placing over 1.3 billion people at risk of being exposed to CHIKV (3). Currently, there are no licensed CHIKV-specific treatments or vaccines to control this disease. Furthermore, the rise in arboviral diseases (such as dengue and chikungunya fever) during the present COVID-19 pandemic is placing an additional strain on already scarce health care resources (4), further stressing the urgent need for effective vaccines against arboviral pathogens, such as CHIKV.

Several promising vaccine candidates against CHIKV are currently in clinical development (5, 6). However, late-stage clinical development has been hampered by the unpredictable nature of CHIKV outbreaks, which requires large participant numbers and long periods of follow-up to demonstrate vaccine efficacy against CHIKV via traditional phase III randomized clinical trials (1). Hence, during the recent 158th Meeting of the Vaccines and Related Biological Products Advisory Committee, it was suggested that data from seroepidemiologic studies and animal challenge studies (passively transferred with human vaccine-immunized sera) can be used to identify an immune correlate predictive of vaccine efficacy (7). Seroepidemiological studies have indicated that a measurable neutralizing antibody titer correlates with protection from symptomatic CHIKV disease (8–11). Passive serum transfer experiments conducted in animals further highlighted the essential role of neutralizing antibodies in the immune defense against CHIKV (12–15).

In addition to direct virus neutralization, virus-specific antibodies can also protect through mechanisms facilitated by the Fc portion binding to Fcγ receptors (FcγR) on immune cells, known as antibody effector functions. Key mechanisms of antibody effector–mediated viral clearance include antibody-dependent cellular phagocytosis (ADCP) and antibody-dependent cellular cytotoxicity (ADCC) (16, 17). A large body of work has highlighted the importance of ADCC and ADCP in the protection against several viral pathogens, such as HIV, influenza, and Ebola viruses (18–21). Recent work on the use of monoclonal antibodies as potential treatment against CHIKV showed that optimal clearance of infection required antibody-FcγR engagement, which mediated enhanced antibody effector mechanisms, such as ADCP (22). Furthermore, potent anti-CHIKV antibodies isolated from individuals with natural infection have been shown to bind CHIKV glycoproteins on the surface of infected cells, preventing both viral egress and initiating ADCC through immune effector cells (23). Therefore, it is important to ensure that protective FcγR-mediated antibody effector functions can also be elicited through CHIKV vaccination.

A wide variety of vaccines for the prevention of chikungunya fever have undergone preclinical evaluation, and several of those have progressed to clinical development (5, 6, 24, 25). MV-CHIK, a measles-vectored, live-attenuated, recombinant vaccine developed by Themis Bioscience GmbH (a wholly owned subsidiary of Merck & Co. Inc.) represents an advanced clinical candidate. This vaccine was strongly immunogenic and protective in animal studies and showed good safety, tolerability, and immunogenicity in phase I and II clinical trials (13, 26, 27). In the phase II clinical trial MV-CHIK-202 (ClinicalTrials.gov NCT02861586; EUDRACT no. 2015-004037-26), administration of 1 or 2 doses of 5 × 10^5^ (±0.5 log) TCID50 of MV-CHIK induced substantial amounts of CHIKV-specific neutralizing antibodies, with the prime-boost (P+B) approach leading to significantly higher antibody titers compared with prime-only (P) vaccination (27). However, it is unclear whether MV-CHIK vaccination can induce protective antibody effector properties. Additionally, it is also unknown whether a P or P+B regimen of MV-CHIK would offer the most protective antibody profile.

In the present study, we aimed to better understand the quality of the humoral immune response induced longitudinally by P and P+B strategies of MV-CHIK vaccination. To this end, a subset of sera collected from the phase II clinical trial MV-CHIK-202 at 1 month or 6 months after vaccination was selected in order to conduct of the 2 arms of the CHKV-specific humoral response, i.e., (a) the Fab antigen-specific properties (specifically antibody quantity, antigen specificity, avidity, and neutralization) and (b) the antibody effector properties (specifically IgG subclass distribution, ADCP, and antibody-dependent NK cell activation [ADNKA] as a proxy for ADCC). We show that MV-CHIK primarily induces IgG1 and IgG3 type antibodies that are functional in the context of ADCP and ADCC. Interestingly, while overall better antibody responses were induced by the P+B approach at 1 month after vaccination, at 6 months after vaccination the P and P+B antibody responses were more similar.

## Results

### P+B with MV-CHIK elicits higher CHIKV-specific antibodies.

**Figure 1:**
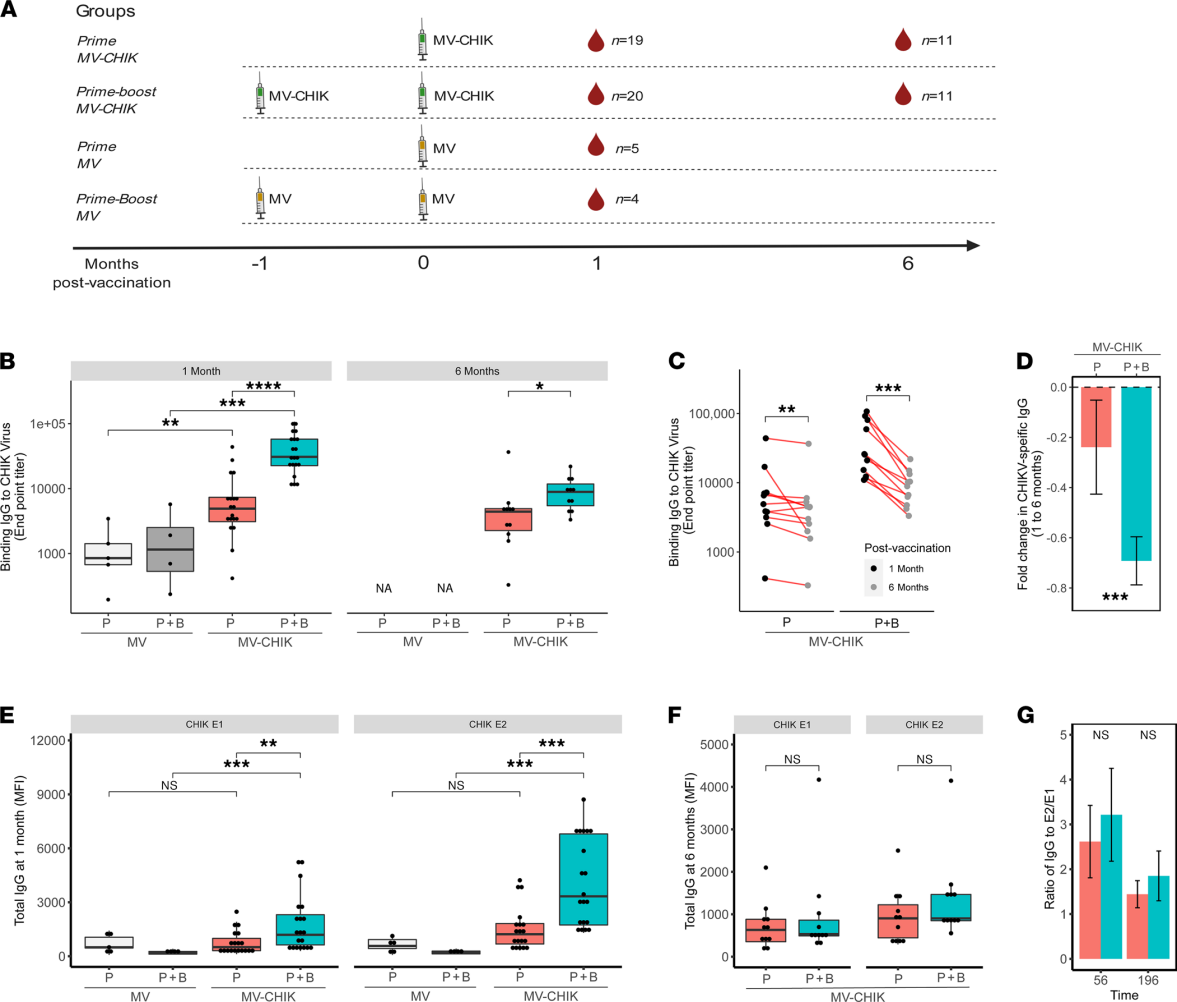
Study design and characterization of the CHIK-specific IgG response elicited by MV-CHIK prime and prime-boost vaccination strategies. (**A**) Participants were vaccinated with either MV-CHIK prime at day 0 (P only) or prime (at month –1) and boost (at day 0) (P+B), and serum antibody responses were compared at 1 and 6 months after vaccination. MV-vaccinated groups served as controls. (**B–F**) Total IgG binding to CHIKV virus was measured using ELISA and compared (**B**) between groups and (**C**) at 1 and 6 months for paired MV-CHIK–vaccinated samples. (**D**) Fold change in IgG (mean ± 95% CI) in response to CHIKV virus was compared between MV-CHIK P only and P+B. IgG binding to 2 CHIK surface glycoproteins, E1 and E2, was measured using Luminex immunoassay and compared between groups at (**E**) 1 month and (**F**) 6 months after vaccination. (**G**) Distribution of the CHIK antigens targeted by the antibody response was estimated by the ratio of E2-specific to E1-specific IgG (mean ± 95% CI) and compared between prime and prime-boost MV-CHIK vaccinations. IgG responses between groups were compared using Mann-Whitney *U* test, while paired analysis between groups at 1 and 6 months was conducted using paired Wilcoxon signed-rank test (*P < 0.05, **P < 0.01, ***P < 0.001, ****P < 0.0001).

Our study compared humoral responses at 1 and 6 months in individuals that were given the MV-CHIK vaccine either as a P or a P+B inoculation about 1 month apart (Figure 1A). Participants who received the licensed measles vaccine, Priorix, served as controls (MV). As expected, MV-CHIK vaccination induced detectable CHIKV-specific IgG antibodies, with IgG titers significantly greater in the MV-CHIK P+B groups as compared with the P group (Figure 1B). CHIKV-specific IgG titers decayed over time (from 1 to 6 months) with a greater fold decrease observed in the P+B MV-CHIK–vaccinated group (i.e., mean fold change ± 95% CI for P was –0.24 ± 0.19 and for P+B was –0.69 ± 0.10) (Figure 1, C and D). We then assessed total IgG binding to the major CHIKV surface glycoproteins, E1 and E2. Similar to previous findings with antibody responses induced by natural CHIKV infections (28), MV-CHIK vaccination predominantly induced antibodies against E2 glycoprotein as compared with E1 (Figure 1, E and F). The MV-CHIK P+B regimen elicited significantly greater IgG responses to E1 and E2 than P (Figure 1, E and F, and Supplemental Figure 1; supplemental material available online with this article; https://doi.org/10.1172/jci.insight.151095DS1). However, the ratio of E2/E1 IgG was similar following both vaccination regimens (Figure 1G), indicating that the boost increased IgG responses against both E1 and E2 evenly and did not change the ratio of antibodies directed against the major antigenic proteins targeted.

### Antibody avidity and CHIKV neutralization following MV-CHIK vaccination.

**Figure 2:**
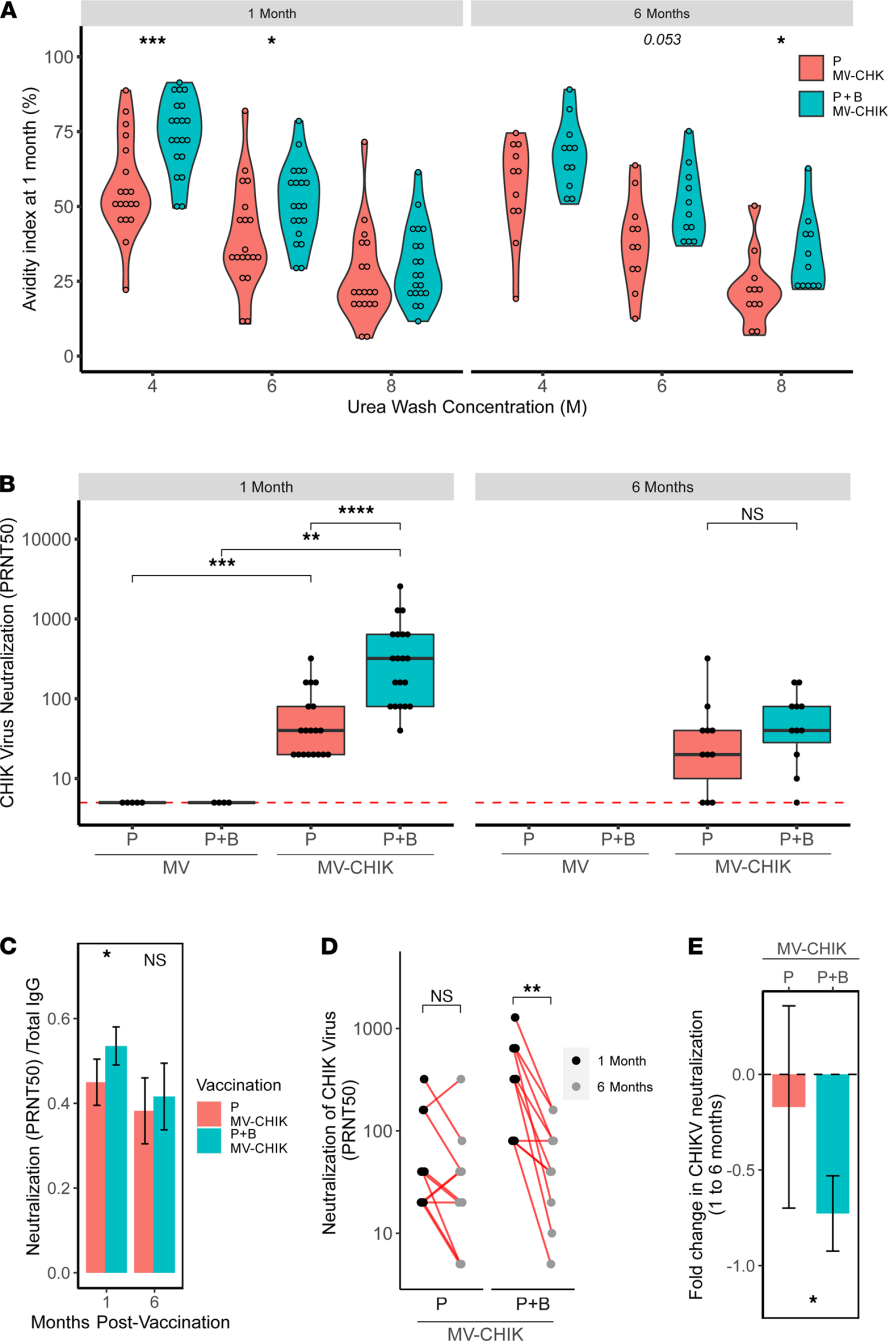
Avidity and neutralizing capacity of CHIK virus–specific antibody responses elicited by MV-CHIK prime and prime-boost immunization. (**A**) Binding strength of CHIK virus–specific antibodies, as indicated by avidity index estimations following 4, 6, and 8 M urea washes. (**B**) CHIK virus neutralization (measured by PRNT assay) and (**C**) neutralization normalized to total IgG (mean ± 95% CI) compared between vaccinated groups. (**D** and **E**) Longitudinal assessment of the CHIKV-neutralizing antibody response using (**D**) paired analysis between 1 and 6 months and (**E**) fold change in CHIKV neutralization from 1 to 6 months (mean ± 95% CI) in MV-CHIK prime (P) and prime-boost (P+B) immunization. Avidity index and PRNT50 titers were compared between groups using Mann-Whitney *U* test, and analysis between groups at 1 and 6 months was conducted using paired Wilcoxon signed-rank test (*P < 0.05, **P < 0.01, ***P < 0.001, ****P < 0.0001).

We then investigated the quality of the CHIKV-specific antibodies induced by MV-CHIK vaccination by assessing both the strength of antibody binding and CHIKV neutralization. Strength of antibody binding was measured as antibody avidity in the presence of 4, 6, and 8 M urea concentrations. In general, the MV-CHIK P+B regimen of vaccination elicited higher avidity antibodies than P, with statistical significance observed in 4 M and 6 M urea at 1 month and in 8 M urea at 6 months (Figure 2A). This observation is expected, as boosting of the humoral immune system leads to affinity maturation of B cells through somatic hypermutations of the variable regions of immunoglobulin genes and selection of higher affinity B cells (29, 30).

CHIKV-neutralizing capacity of antibodies elicited by MV-CHIK vaccination was measured using a plaque reduction neutralization test (PRNT). As observed previously, MV-CHIK vaccine elicited potently neutralizing antibodies (27). Neutralization titers for the subset of samples used in the present study are displayed in Figure 2. The P+B regimen resulted in significantly greater neutralization than P vaccination at 1 month after vaccination (Figure 2, B and C). Interestingly, longitudinal analysis of CHIKV neutralization showed significantly greater waning of neutralizing antibodies between 1 and 6 months following P+B MV-CHIK vaccination as compared with P vaccination (Figure 2, D and E). Despite the lack of statistically significant difference, the P+B MV-CHIK–vaccinated group at 6 months did trend toward higher neutralization. Altogether, these data suggest that (for the subset of samples used in the current study) the P+B MV-CHIK–vaccinated individuals displayed significantly higher IgG, avidity, and transiently higher neutralization.

### MV-CHIK vaccine elicits a dominant IgG3 response.

**Figure 3:**
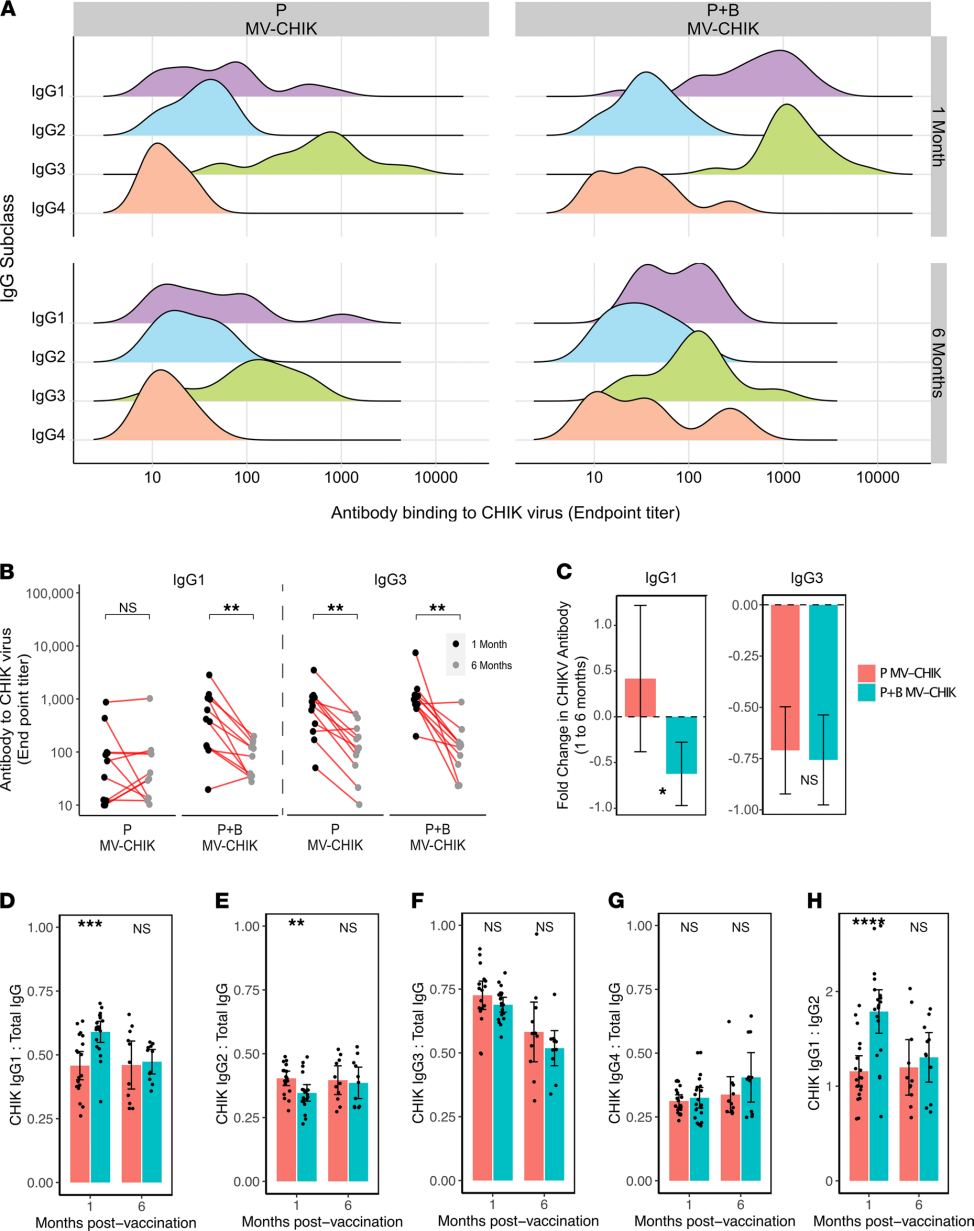
IgG subclass distribution of CHIK virus–specific antibody responses following MV-CHIK prime and prime-boost vaccinations. (**A**) IgG1, IgG2, IgG3, and IgG4 CHIK virus–binding endpoint titers (measured by ELISA) in MV-CHIK–vaccinated individuals. (**B** and **C**) Longitudinal analysis of CHIK virus–specific IgG1 and IgG3. (**B**) Endpoint titers at 1 and 6 months. (**C**) Fold change in antibody titers from 1 to 6 months (mean ± 95% CI). (**D–G**) The ratio of each IgG subclass (IgG1, IgG2, IgG3, and IgG4) to total IgG, and (**H**) the ratio of IgG1 to IgG2 (mean ± 95% CI). IgG subclass titers were compared between groups using Mann-Whitney *U* test, and comparisons between groups at 1 and 6 months used paired Wilcoxon signed-rank test (**P < 0.01, ***P < 0.001, ****P < 0.0001).

Antibody responses to viruses can protect through both antigen-specific processes mediated by the Fab portion (such as neutralization) and through FcγR-mediated antibody effector functions through the antibody Fc portion (such as ADCP and ADCC). Attributes, such as the subclass of IgG, can affect both antibody effector functions and neutralization (31, 32). Of note, in the case of CHIKV natural infections, IgG3 has been shown to be the dominant IgG subclass responsible for neutralization; it has even been proposed as a possible early marker of protection (33, 34). Therefore, we explored the generation of IgG subclasses following MV-CHIK vaccination. Similar to natural infections, IgG3 was the major virus-binding IgG subclass generated following MV-CHIK vaccination (median IgG3 titers at 1 month for P and P+B were 626 [IQR 287–1068] and 1146 [IQR 900–1818], respectively), and this trend persisted even at 6 months (Figure 3A and Supplemental Figure 2). In addition to IgG3, boosting generated significant levels of CHIK virus–specific IgG1 at 1 month after vaccination, which rapidly waned over time (median IgG1 titers at 1 and 6 months were 641 [IQR 267–1085] and 83 [IQR 35–135], respectively) (Figure 3, A–C, and Supplemental Figure 2). Interestingly, the fold change in IgG3 over time was similar following both vaccination regimens (i.e., –0.71 ± 0.21 and –0.76 ± 0.22 for P and P+B, respectively) (Figure 3C). Assessment of subclasses of IgGs against the 2 CHIKV glycoproteins, E1 and E2, further confirmed that IgG3 and IgG1 were the dominant antibody subclasses produced following MV-CHIK vaccination (Supplemental Figure 3). We then explored whether a boost would skew the distribution of certain IgG subclasses. Apart from IgG1/total IgG and IgG2/total IgG at 1 month after vaccination, there were no significant differences in the ratios of IgG subclasses between P and P+B vaccine regimens (Figure 3, D–G). Additionally, IgG1/IgG2 ratios were greater than 1 (Figure 3H), suggesting that MV-CHIK vaccine elicits Th1 dominant responses.

### Vaccination with MV-CHIK induces antibodies capable of ADCP.

**Figure 4:**
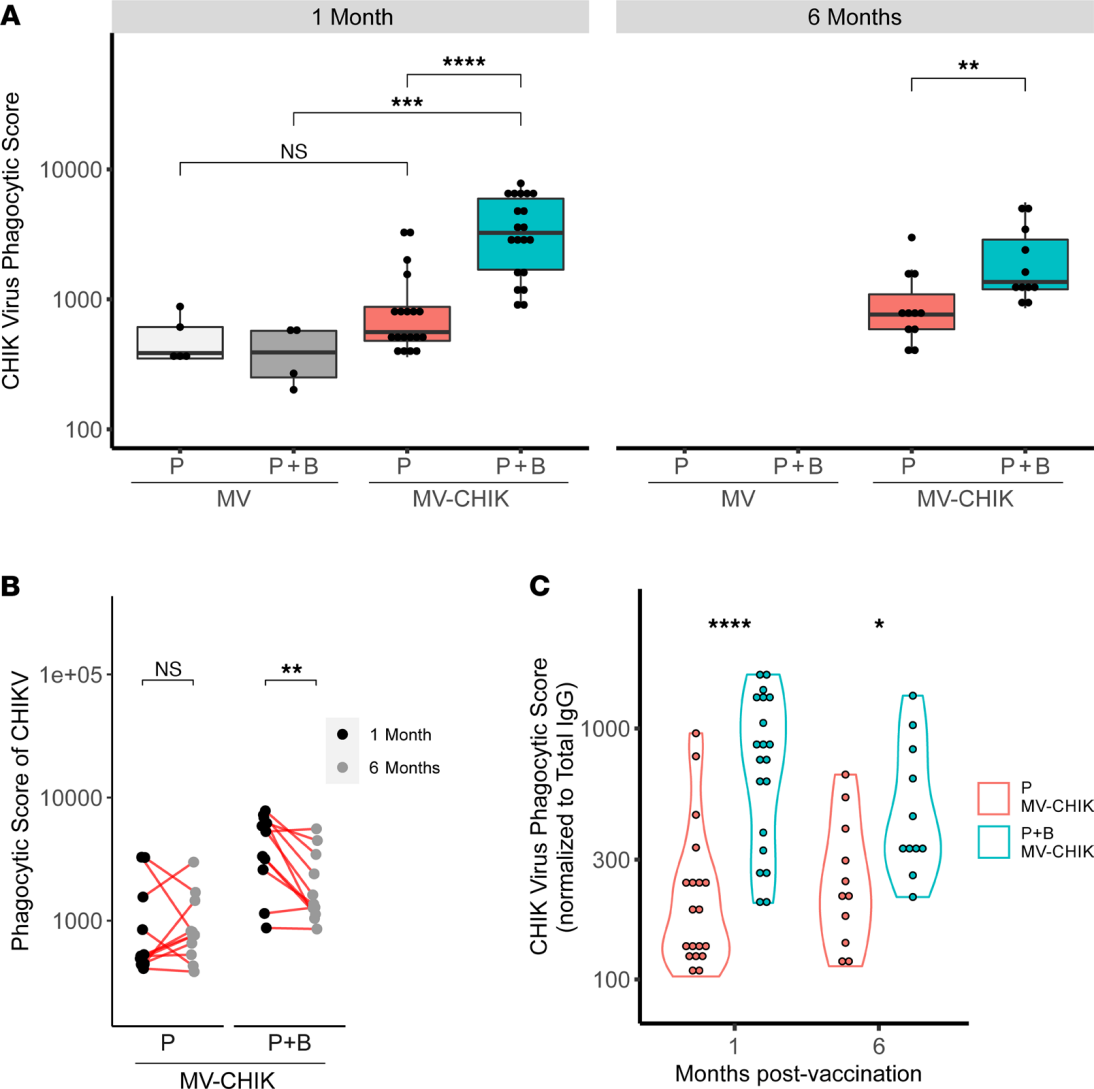
Antibody-dependent cellular phagocytosis of CHIK virus by a monocytic cell line following vaccination with MV-CHIK. (**A**) Phagocytic scores (measured using a whole-virus phagocytic assay) compared between vaccinated groups. (**B**) Paired longitudinal antibody-dependent cellular phagocytosis (ADCP) responses compared at 1 and 6 months. (**C**) Normalized phagocytic scores (i.e., phagocytic scores normalized to CHIKV-specific IgG titers) compared between P- and P+B MC-CHIK–vaccinated groups. Phagocytic scores and normalized phagocytic scores were compared between groups using Mann-Whitney *U* test, and paired analysis between groups at 1 and 6 months was conducted using paired Wilcoxon signed-rank test (*P < 0.05, **P < 0.01, ***P < 0.001, ****P < 0.0001).

We then investigated the ADCP potential of CHIKV-specific antibodies induced by MV-CHIK. At 1 month, the phagocytic score obtained after MV-CHIK P vaccination was not significantly greater than that of the MV controls; however, the phagocytic score was substantially higher following the P+B MV-CHIK vaccination regimen (median phagocytic scores for P and P+B at 1 month were 560 [IQR 480–875] and 3250 [IQR 1698–5953], respectively) (Figure 4A). Despite a decrease in phagocytic score between 1 and 6 months (Figure 4B), it remained higher in the P+B–vaccinated group than in the P-vaccinated group at 6 months (Figure 4A). Furthermore, despite normalization of ADCP activity to CHIKV-specific IgG, the P+B MV-CHIK–vaccinated individuals maintained a greater ADCP activity (Figure 4C). This indicates that, in addition to higher CHIKV-specific IgG, there may be other yet unmeasured antibody effector properties that promote higher ADCP activity following P+B MV-CHIK vaccination.

### MV-CHIK vaccination elicits antibodies capable of antibody mediated NK degranulation and cytokine release.

**Figure 5:**
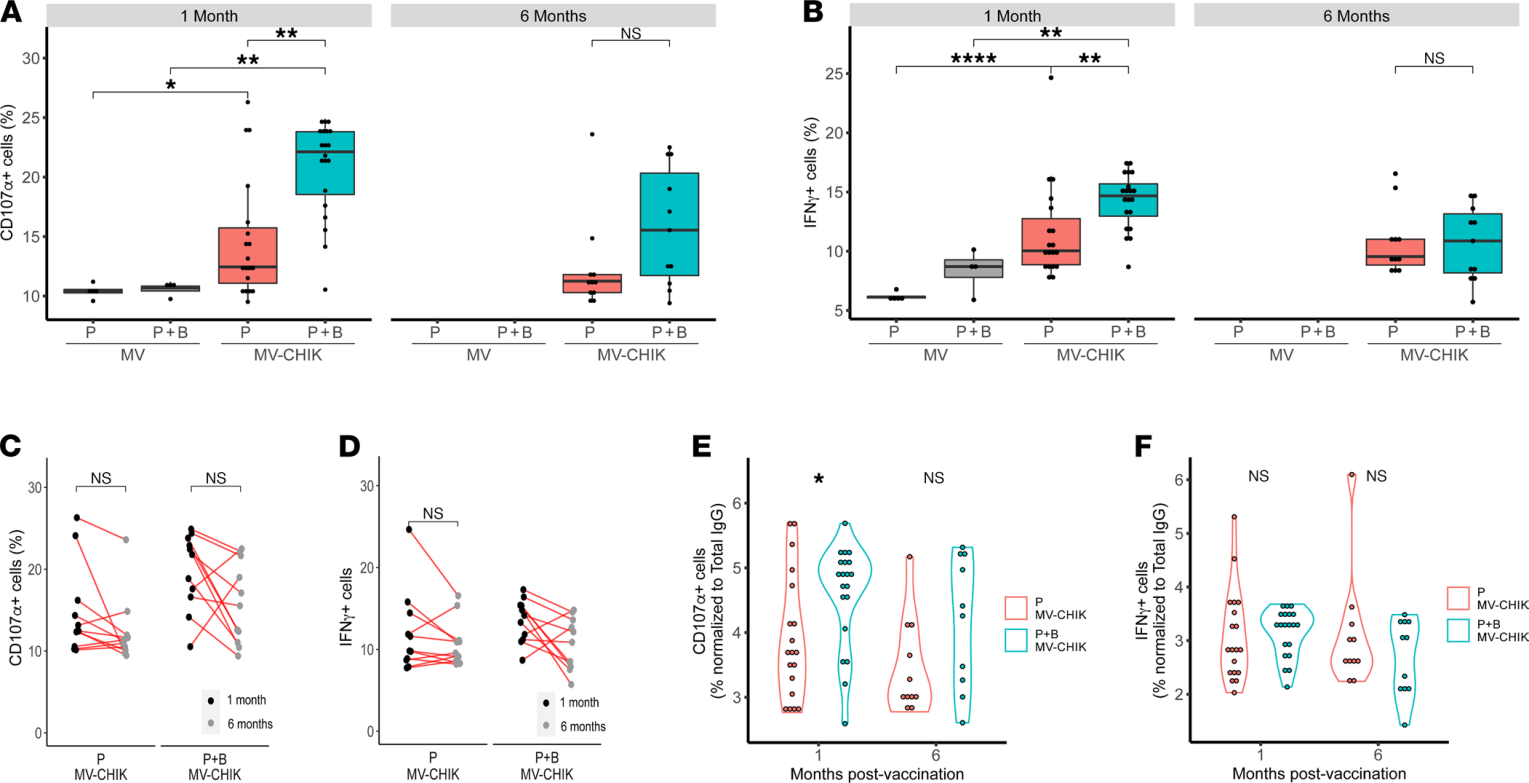
NK-mediated ADCC following MV-CHIK vaccination. ADCC was assessed through the proxy measures of ADNKA degranulation (CD107α) and cytokine release (IFN-γ). (**A**) Percentage of CD107α^+^ cells and (**B**) IFN-γ^+^ cells in vaccinated groups. Paired longitudinal analysis of the percentage of (**C**) CD107α^+^ cells and (**D**) IFN-γ^+^ cells at 1 and 6 months after vaccination. Normalized percentage of (**E**) CD107α^+^ cells and (**F**) IFN-γ^+^ cells (to CHIKV-specific IgG titers) in P- and P+B MV-CHIK–vaccinated groups. Percentages and normalized percentages of CD17α^+^ and IFN-γ^+^ were compared between groups using Mann-Whitney *U* test, and paired analysis between groups at 1 and 6 months was conducted using paired Wilcoxon signed-rank test (*P < 0.05, **P < 0.01, ***P < 0.001, ****P < 0.0001).

Using ADNKA as a proxy measurement, we further assessed the capacity of MV-CHIK vaccine–induced antibodies to initiate NK-mediated ADCC. MV-CHIK vaccination elicited significant ADNKA, measured by the degranulation marker CD107α (Figure 5A) and cytokine IFN-γ (Figure 5B), respectively. As expected, the P+B MV-CHIK vaccination regimen led to more ADNKA than P vaccination (the percentage of CD107α^+^ cells at 1 month for P and P+B was 22 [IQR 19–24] and 12 [IQR 11–16], and the percentage of IFN-γ^+^ cells was 15 [IQR 13–16] and 10 [IQR 9–13], respectively) (Figure 5A). No statistically significant longitudinal changes in CD107α^+^ cells and IFN-γ^+^ cells were observed between 1 and 6 months (Figure 5, E and F). Normalization of percentages of CD107α^+^ cells (Figure 5C) and IFN-γ^+^ cells (Figure 5D) to IgG removed these major differences between P and P+B vaccination regimens, highlighting that differences in ADCC (specifically NK degranulation and cytokine release) may largely be attributable to differences in CHIKV-specific IgG between the 2 vaccine regimens.

### MV-CHIK P versus P+B vaccinations produce similar long-term humoral responses.

**Figure 6:**
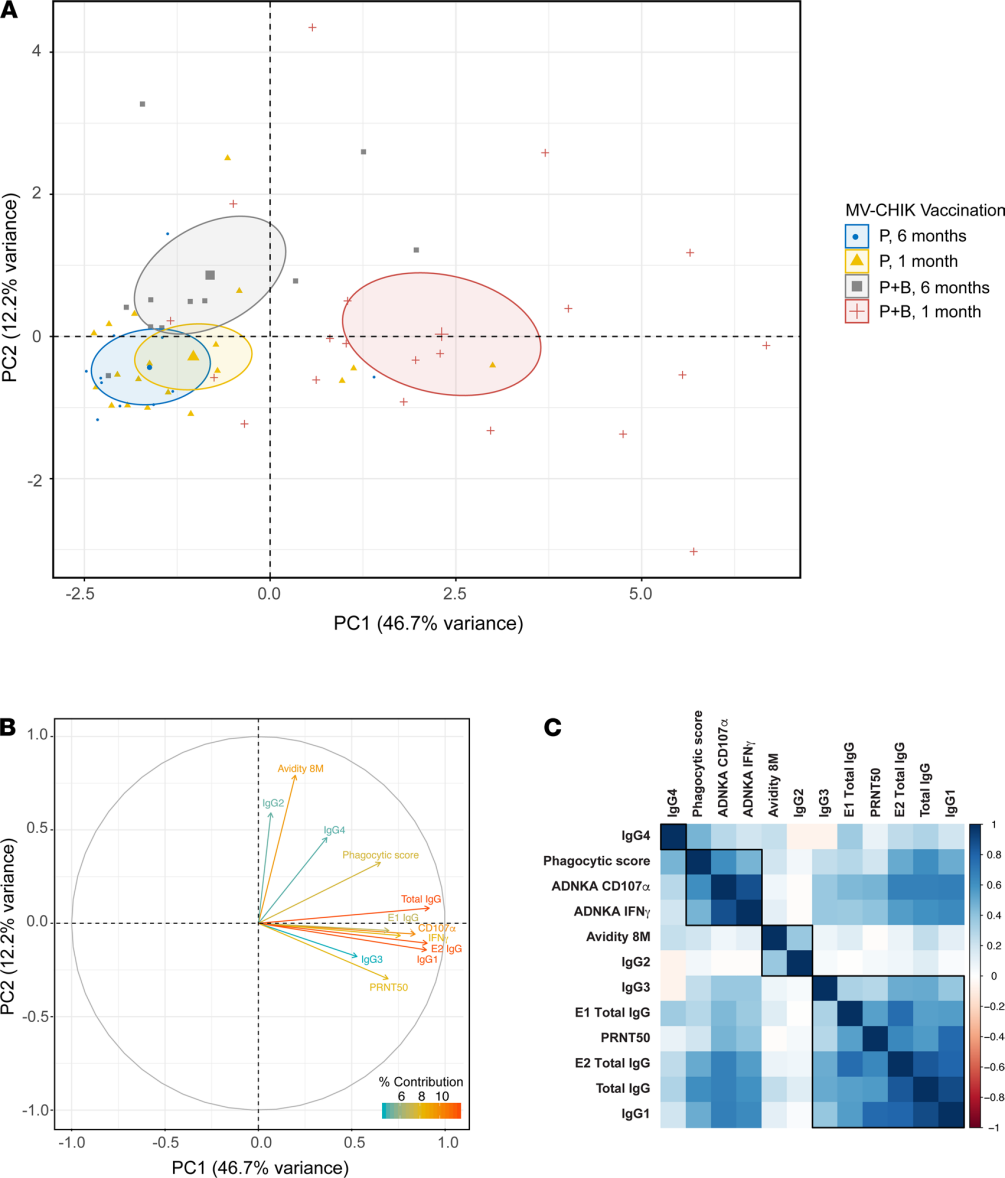
Multidimensional unsupervised analysis of MV-CHIK–vaccinated groups and measured antibody phenotypes. (**A** and **B**) Principal component analysis (PCA) was conducted on MV-CHIK vaccine–induced antibody responses following prime (P) and prime-boost (P+B) regiments at 1 and 6 months. (**A**) Individual plot with vaccine groups highlighted in different colors. Ellipses in the PCA individual plot represent the confidence ellipses around the centroid (marked by the larger symbol for each group) of each vaccine group. (**B**) Circle plot highlighting the variables contributing to the spread of points. (**C**) Correlation matrix with hierarchical clustering of variables utilized in the PCA analysis. Variables included in the PCA and hierarchical correlation matrix include neutralization (PRNT50 titers); virus-binding total IgG, IgG1, IgG2, IgG3, and IgG4 (endpoint titers); E1-binding IgG (MFI); E2-binding IgG (MFI); avidity at 8 M (AI); ADCP (phagocytic score); and ADCC (ADNKA degranulation as percentage of CD107α^+^ cells, and ADNKA cytokine release as percentage of IFN-γ^+^ cells).

Composite antibody profiles of the MV-CHIK–vaccinated groups were assessed using the multidimensional analysis technique principal component analysis (PCA). PCA is a dimension reduction technique that represents multidimensional data graphically to allow for easy detection of patterns within the entire data set. Current PCA analysis was conducted using the following variables: PRNT50 titers; virus-binding total IgG, IgG1, IgG2, IgG3, and IgG4; E1-binding IgG; E2-binding IgG; avidity at 8 M; ADCP; ADNKA degranulation (percentage of CD107α^+^ cells); and ADNKA cytokine release (percentage of IFN-γ^+^ cells). The individual plot (Figure 6A) represents the vaccinated individuals on the PCA plane, colored by vaccination regimen P and P+B at 1 month. At 1 month after vaccination, P- and P+B–vaccinated groups showed no overlap on the PCA plot, indicating that the antibody profiles immediately after vaccination were significantly different between the 2 vaccination regimens (Figure 6A). As indicated by the variable circle plot, these short term (i.e., at 1 month) differences between the vaccination groups are associated with P+B eliciting higher virus-binding total IgG, IgG1, and IgG3; E1-binding IgG; E2-binding IgG; PRNT50 titers; ADCP; and ADCC (ADNKA degranulation, and cytokine release) (Figure 6, A and B). Of these variables, the hierarchical correlation matrix showed that several antibody variables positively correlated with each other, including (a) ADCP (phagocytic score) and ADCC (as measured by ADNKA CD107α and IFN-γ), (b) avidity and IgG2, and (c) E1-IgG, PRNT50, E2-IgG, and total IgG and IgG1 (Figure 6C). Additionally, we also observed that at 1 month, differences in the quantity of vaccine-induced CHIKV-specific IgG alone were not significantly associated with differences in antibody effector properties and functions (Supplemental Figure 4). Furthermore, additional correlation analyses with baseline measles antibody titers showed that baseline measles-specific binding IgG titers did not significantly influence any of the measured CHIK-specific antibody responses in either MV-CHIK–vaccinated groups (Supplemental Figure 5).

Temporal antibody profiles following P and P+B MV-CHIK–vaccinated groups were also compared. A fair overlap between the P groups at 1 and 6 months was observed in the PCA, indicating that the antibody profiles in the P group do not change significantly within the first 6 months (Figure 6A). However, in the P+B vaccination group, PCA analysis showed major changes in antibody profiles between 1 and 6 months. In fact, the antibody profiles of the P+B vaccine group at 6 months were similar to those of the P vaccine group, with the major differentiating factors being ADCP and antibody avidity (Figure 6, A and B). These observations led us to conclude that, despite large differences between the 2 vaccine regimens in the short term, they both lead to fairly similar antibody profiles in the long-term.

## Discussion

In this study, we characterized both Fab- and Fc-mediated functions of antibodies induced by the CHIKV vaccine candidate MV-CHIK and assessed potential differences between P+B and P vaccine administration regimens. Preclinical work in mice and NHPs has shown that MV-CHIK induces neutralizing antibodies and protects animals from both acute disease and chronic sequelae (13, 35). Several phase I and II clinical trials have confirmed that substantial levels of neutralizing antibodies are also induced in humans upon MV-CHIK immunization (1, 26, 35). In line with published immunogenicity data in the MV-CHIK-202 trial (27), the present study observed that CHIKV-specific binding and neutralizing antibody titers were significantly higher soon after a PB immunization as compared with a P regimen. Additionally, we showed that these boosted antibody responses bound CHIKV virus with greater avidity. These observations mirror typical secondary antibody responses, where a second vaccine dose elicits rapid expansion of memory B cells with somatic hypermutations and thereby results in an antibody pool of greater specificity, affinity, and neutralization (36–38). Surface CHIKV glycoprotein E2 has been documented to be the major target of neutralizing human antibodies following natural CHIKV infections (28, 34, 39–41). Similarly, the MV-CHIK vaccine elicited mostly antibodies that bound E2 antigen (42), and antigen specificity was not affected by administration regimen.

Despite the importance of Fc-mediated antibody effector functions in protecting against CHIKV infections, current CHIKV vaccine candidates under development have mostly been characterized for their potential to elicit antibodies that are neutralizing (24, 25, 27). To address this knowledge gap, our study characterized antibody effector properties (such as IgG subclasses) and functions (such as ADCP and ADCC) induced by the MV-CHIK vaccine. IgG subtyping in the present study revealed that both administration regimens of MV-CHIK vaccine induced substantial amounts of IgG3. Interestingly, the early appearance of neutralizing IgG3 antibodies following natural CHIKV infections has been correlated with virus clearance and protection from chronic disease (33). Consequently, we observed that MV-CHIK vaccine was able to induce strong antibody effector functions, such as ADCC (specifically ADNKA degranulation and cytokine release) and ADCP (through monocytic cells). Potent effector functions were probably a result of a predominantly IgG3 response, as IgG3 is the subclass typically known to induce FcγR-mediated removal of virus and infected cells (43). Differences in ADCC functions between the two vaccine administration regimens were only transient, indicating that these differences were probably directly influenced by the differences in the quantity of antibody that subsequently decreased over time. This observation prompted us to compare qualitative aspects of the immune response in participants having received either 1 or 2 doses of MV-CHIK vaccine stratified by binding IgG antibody titers. Once adjusted for antibody quantity, we found all parameters (except for ADCP) to be remarkably similar between high and low IgG titer groups.

In addition to IgG subclass, antibody effector functions are also influenced by the type of glycosylation on the Fc portion (44, 45). The type of glycosylation on an antibody Fc region is determined during B cell activation and differentiation by inflammatory factors, such as cytokines and the immune environment (46). Since prime and booster vaccination usually elicit widely different cytokine and innate immune responses (47), it is foreseeable that the booster doses would generate antibody responses with varying Fc glycosylations. Past studies have observed that repeated immunization with the same antigen can lead to greater IgG Fc fucosylation (48). Therefore, it is possible that the long-term differences in ADCP between the P and P+B MV-CHIK vaccine regimens are a consequence of differences in both antibody quantity and glycosylation. Although significant long-term differences in ADCP and avidity between the administered vaccine regimens were noted, it is unclear how these differences in antibody parameters relate to protection against CHIKV infections. Therefore, because traditional phase III efficacy randomized controlled trials are not feasible for CHIKV vaccine testing, future NHP passive antibody transfer and virus challenge experiments could be accompanied by a systems serology level antibody characterization to enable discovery of additional humoral immune correlates of protection.

In conclusion, the current study is the first characterization to our knowledge of both antigen-specific– (Fab-) and antibody effector–mediated (Fc-mediated) functions comparing P and P+B regimens of CHIKV vaccination. Clinical trials with the CHIKV vaccine, MV-CHIK, indicate that PB administration (as opposed to P) induces higher, more durable levels of protective neutralizing antibodies (26, 27). Together with the data presented here, this suggests that a booster MV-CHIK dose induces more potent antibody responses in terms of both Fab -and Fc-mediated protective mechanisms. Therefore, P+B regimen for MV-CHIK presents itself as the preferred regimen for achieving long-term protection in CHIKV endemic situations. However, because several other antibody parameters highlighted a surprising similarity in the humoral response induced by both vaccine regimens at 6 months, the P regimen could be extremely useful in a situation in which a rapid and simple immunization strategy is necessary to curb virus spread, such as during a local virus outbreak. Similarly, a 1-dose regimen might mediate the short-term protection required for travelers going to CHKV-endemic regions. Therefore, we recommend future postlicensure studies to also investigate protection by CHIKV vaccines in 1- versus 2-dose immunizations.

## Methods

### Volunteers and vaccination.

Sera from healthy volunteers enrolled in the phase II clinical study MV-CHIK-202 (ClinicalTrials.gov NCT02861586; EUDRACT no. 2015-004037-26) was collected at various time points as previously described (27). Participants received either Priorix, a licensed MMR vaccine containing the parental measles Schwarz strain (MV), used as a comparator, or MV-CHIK, a vaccine candidate for the prevention of chikungunya fever, at a dose of 5 × 10^5^ TCID50 per administration.

### Antibody-binding measurements using ELISA.

Binding of serum IgG and IgG subclasses (i.e., IgG1, IgG2, IgG3, and IgG4) to CHIKV virions was assessed using an in-house indirect ELISA. CHIKV clinical isolate from Singapore (SG 009/06, Indian Ocean lineage) was grown in BHK-21 cells (ATCC), and virus-containing supernatant was harvested and virus purified using a 30% sucrose cushion. Purified CHIK virus (100 ng/well) was coated on NUNC Maxisorp ELISA plates (Thermo Fisher Scientific) and incubated overnight at 4°C. Plates were washed with 0.2% PBS-T (i.e., 0.2% Tween20 in 1X PBS) and blocked with 1% casein (1 hour at 37°C), followed by incubation with serially diluted serum samples for 1 hour at 37°C. For IgG endpoint titrations, serum tested at 4-fold dilutions ranging from 1:25 to 1:102,400. For IgG subclass measures, serum was tested at 3-fold dilutions ranging from 1:10 to 1:7290. ELISA plates were then washed and probed with the relevant HRP-conjugated secondary antibody for detection of human IgG (Santa Cruz, sc-2907) and IgG1, IgG2, IgG3, and IgG4 (Southern Biotech, 9054-05, 9060-05, 9210-05, and 9200-05, respectively). Plates were developed with 3,3′,5,5′-tetramethylbenzidine (TMB) substrate (Seracare), development stopped with 1 M HCl, and absorbance (OD 405 nm) recorded using a spectrophotometer (Varioskan Lux, Thermo Fisher Scientific). A 4-parameter logistic regression curve was fitted to the measured data, and average background signals plus 4 standard deviations was set as the threshold cutoff to estimate endpoint titers. The ratio of each IgG subclass (IgG1, IgG2, IgG3, and IgG4) to total IgG was computed by dividing log_10_-transformed IgG subclass titers with log_10_-transformed CHIKV-specific IgG endpoint titers.

Measles virus-specific IgG antibodies were quantified using a commercially available ELISA kit (Euroimmun), according to the manufacturer’s instructions.

### Avidity assay.

Antibody avidity was estimated using modified ELISA assays as previously described for other viruses (49). Briefly, purified CHIKV-coated ELISA plates were blocked and probed with human serum (diluted at 1:25) for 1 hour at 37°C. Each probed sample was then incubated for 10 minutes at room temperature with either wash buffer (i.e. 0.2% PBS-T) or urea (4 M, 6 M, or 8 M). Plates were then washed 3 times, probed with anti-human IgG-HRP (1 hour at 37°C), washed 3 times, and developed with TMB substrate; absorbance was read at OD 405 nm. Avidity index was calculated as follows:

   Equation 1.
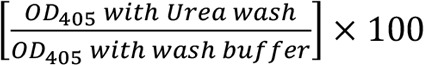


### Antibody-binding measurements using multiplex immuno-Luminex assay.

Binding of antibody to recombinant CHIK E1 and E2 glycoproteins (Aalto Bio, BM6300 and BM 6284, respectively) was assessed using multiplex immuno-Luminex assays (similar assays have been published previously, ref. 50). Briefly, recombinant E1 and E2 proteins were covalently conjugated to 2 different bead colors of Luminex MagPlex-C microspheres (at saturating concentrations, i.e., 5 g/ million beads) using carboxyl chemistry (with the Luminex xMAP ABC coupling kit, as per manufacturer protocol). Conjugated beads (1250/well) were added to 96 well U-bottom plates and blocked with block buffer (i.e., 1% BSA in PBS) for 30 minutes at 37°C (while shaking at 250 rpm). Sera was then diluted 1:100 in block buffer and incubated with blocked beads for 1 hour at 37°C (while shaking), following which beads were washed 3 times using an automated magnetic bead washer (BioTek, 50S). Antigen-specific IgG, IgG1, IgG2, IgG3, and IgG4 were then probed with the secondary antibodies, anti-human IgG-PE (Invitrogen, 12-4998-82), anti-human IgG1-Biotin Maleimide (Southern Biotech, 9054-28), anti-human IgG2-biotin (Southern Biotech, 9060-08), anti-human IgG3-biotin (Southern Biotech, 9210-08), and anti-human IgG4-biotin (Abcam, ab99818). Binding of biotinylated secondary antibodies was then detected using PE-conjugated streptavidin (Biolegend, 405204). Antibody binding data were collected using a Magpix instrument, as median fluorescence intensity (MFI) per bead.

### PRNT.

Neutralizing antibodies were measured using a PRNT performed as published previously (26). In brief, serial dilutions of serum were incubated with the LR-2006-OPY isolate of CHIKV for 1 hour. This mixture was added to monolayers of Vero cells (ATCC, CCL-81) cultured in 6-well plates. Plates were incubated for 1 hour to allow for infection, followed by agarose overlay. Plates were again incubated for 48 hours and stained with Crystal Violet overnight, followed by plaque counting. The PRNT50 value was defined as the dilution reducing the number of plaques to less than 50% of the control value (i.e., cells infected with virus only). Normalized PRNT50 titers were calculated by dividing log_10_-transformed PRNT50 titers by log_10_-transformed CHIKV-specific IgG endpoint titers.

### ADCP.

ADCP assays were conducted using virus labeled with a pH-sensitive dye called pHrodo (as has been used to study viral entry in previous studies, ref. 51). Purified CHIKV virus was incubated for 45 minutes at room temperature with 50 μM pHrodo Red, succinimidyl ester (Thermo Fisher Scientific, P36600) in 0.1 M bicarbonate buffer. Excess dye was removed by passing labeled virus through a G-50 micro-spin column (GE Healthcare). Labeled CHIK virus was incubated with serum samples (diluted at 1:100 in cell culture media) for 1 hour at 37°C under 5% CO_2_. Virus-antibody complexes were then added to a human monocytic cell line, THP-1 (ATCC TIB-202), and incubated for 5 hours at 37°C under 5% CO_2_. Following incubation, cells were washed with 1X PBS, fixed with 4% PFA, and resuspended in FACS buffer (2% FBS, 0.4% 0.5 M EDTA in 1X PBS). The percentage and MFI of virus^+^ cells were assessed using flow cytometry (BD LSR Fortessa). Representative FACS plots for ADCP with no immune sera and with MV-CHIK–vaccinated immune sera are presented in Supplemental Figure 6A. Phagocytic scores were calculated by multiplying the percentage of cells positive for viral entry with the MFI of those positive cells. Normalized phagocytic scores were calculated by dividing ADCP phagocytic scores by log_10_-transformed CHIKV-specific IgG endpoint titers.

### ADNKA assay.

ADCC of CHIKV was assessed with a proxy measurement using a slightly modified NK cell degranulation or ADNKA assay as described previously (18). Briefly, NUNC Maxisorp ELISA plates were washed with sterile 0.1 M bicarbonate buffer and coated with 200 ng/well of purified CHIK virus (2 hours at room temperature). Plates were then washed 3 times with sterile 1X PBS and blocked overnight at 4°C with 5% BSA in 1X PBS. Blocked plates were incubated with diluted serum samples (1:100 in 1% BSA buffer) for 1 hour at 37°C. Washed plates were then incubated with the NK92.CD16 cell line (ATCC, PTA-6967) containing 20 μg/ml Brefeldin A (MilliporeSigma, B7651), protein transport inhibitor (GolgiStop, BD 554724), and anti-CD107α-BV421 antibody (BD, 562623) in the cell culture media for 5 hours at 37°C in 5% CO_2_. Following incubation, cells were fixed and permeabilized with Cytofix/Cytoperm (BD, 554714), stained for IFN-γ (anti–IFN-γ-APC, BD, 554702), and washed, and the percentage of NK cells producing CD107α and IFN-γ was acquired using flow cytometry (BD LSRFortessa). Representative FACS plots for ADNKA CD107α and IFN-γ with no immune sera and with MV-CHIK–vaccinated immune sera are presented in Supplemental Figure 6, B and C, respectively. Percentages of CD107α^+^ cells or IFN-γ^+^ cells were divided by log_10_-transformed CHIKV-specific IgG endpoint titers to estimate normalized percentage of CD107α^+^ and IFN-γ^+^ cells, respectively.

### Data analyses.

PCA was performed to describe the multidimensional serum profiles of the different vaccine groups using the R software packages FactoMineR (52) and factoextra (53). The following variables were scaled to unit variance and included in the analysis: neutralization (PRNT50 titers); virus-binding total IgG, IgG1, IgG2, IgG3, and IgG4 (endpoint titers); E1-binding IgG (MFI); E2-binding IgG (MFI); avidity at 8 M (AI); ADCP (phagocytic score); ADCC degranulation (percentage of CD107^+^ cells); and ADCC cytokine release (percentage of IFN-γ^+^ cells). Subsequently, the individuals on the individual plot were colored according to their group, including the barycenter (mean point) of each along with the 95% confidence ellipse around it. A variable correlation circle plot was also generated for the antibody parameters analyzed in the PCA. A correlation matrix with hierarchical clustering was generated using the R software package corrplot (54) and the same variables used in the PCA.

### Statistics.

Statistical analyses of measured antibody parameters were compared between vaccinated groups using the nonparametric statistical test, Mann-Whitney *U* test. All paired comparisons of measured antibody parameters between 1 and 6 months after immunization within the same individuals were conducted using paired Wilcoxon signed-rank test. All graphing and statistical analyses were conducted using the R software v3.5.1 (55) and package ggplot2 (56). *P* values of less than 0.05 were considered significant.

### Study approval.

Sera used in this study were obtained during clinical trial MV-CHIK-202 (ClinicalTrials.gov NCT02861586; EudraCT 2015-004037-26). MV-CHIK-202 was performed in accordance with Good Clinical Practice guidelines, the Declaration of Helsinki, and all applicable national laws. The protocol was approved by the lead ethics committees of Vienna (approval no. 1957/2015) and the state of Berlin (approval no. 15/0502-Ek15) as well as by the local ethics committees of each study center (Department of Clinical Pharmacology and Institute of Specific Prophylaxis and Tropical Medicine, Medical University of Vienna, Vienna, Austria; Hansa Sanatorium Graz, Graz, Austria; and Department of Tropical Medicine and Infectious Diseases, Rostock University Medical Center, Rostock, Germany.). Written informed consent was obtained from all participants before entry into the study and covered the future use of study samples for research purposes.

## Author contributions

RT, RMZ, KR, and RDA conceived and designed the study. MJK, YSL, and RDA performed the experiments and generated the data. RT, RMZ, and RDA conducted the data and statistical analysis. RT, RMZ, KR, and RDA wrote the manuscript, and CWM, JGL, and EEO also reviewed the data and manuscript.

## Supplementary Material

Supplemental data

## Version 1 09/28/2021

In-Press Preview

## Version 2 11/08/2021

Electronic publication
