# Supplementary material for: Antibody effector analysis of prime versus prime-boost immunizations with a recombinant measles-vectored chikungunya virus vaccine
Source: JCI Insight. 2021 Nov 8;6(21):e151095. doi: 10.1172/jci.insight.151095 (PMC8663552; doi:10.1172/jci.insight.151095)
Supplement: Supplemental data [file jciinsight-6-151095-s205.pdf]

Supplementary Figures

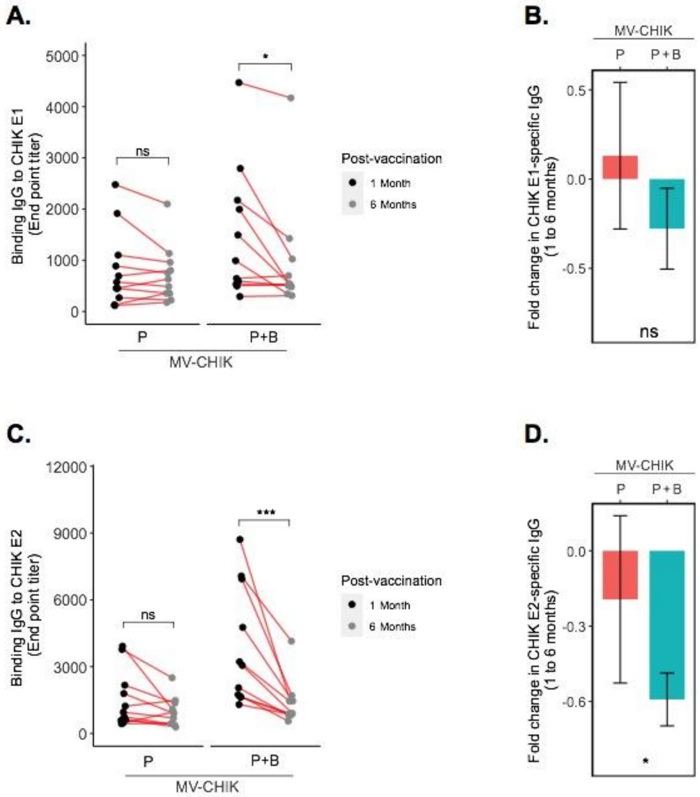

Supplementary Figure 1. Longitudinal IgG responses to CHIK E1 and E2 following vaccination with MV-CHIK prime and prime-boost regiments. CHIK E1-specific IgG responses using (**A**) paired analysis between 1 and 6 months and (**B**) fold change in CHIK E1-specific IgG (mean ± 95%CI) from 1 to 6 months in MV-CHIK prime and prime-boost groups. Similarly, CHIK E2-specific IgG responses compared (**C**) over time using paired analysis and (**D**) in MV-CHIK prime and prime-boost using fold change (mean ± 95%CI) from 1 to 6 months. Fold changes in IgG responses were compared between groups using Mann-Whitney *U* test, and paired analysis between 1 and 6 months conducted using paired Wilcoxon-signed rank test (*P*-values ****<0.0001, ***<0.001, **<0.01, *<0.05).

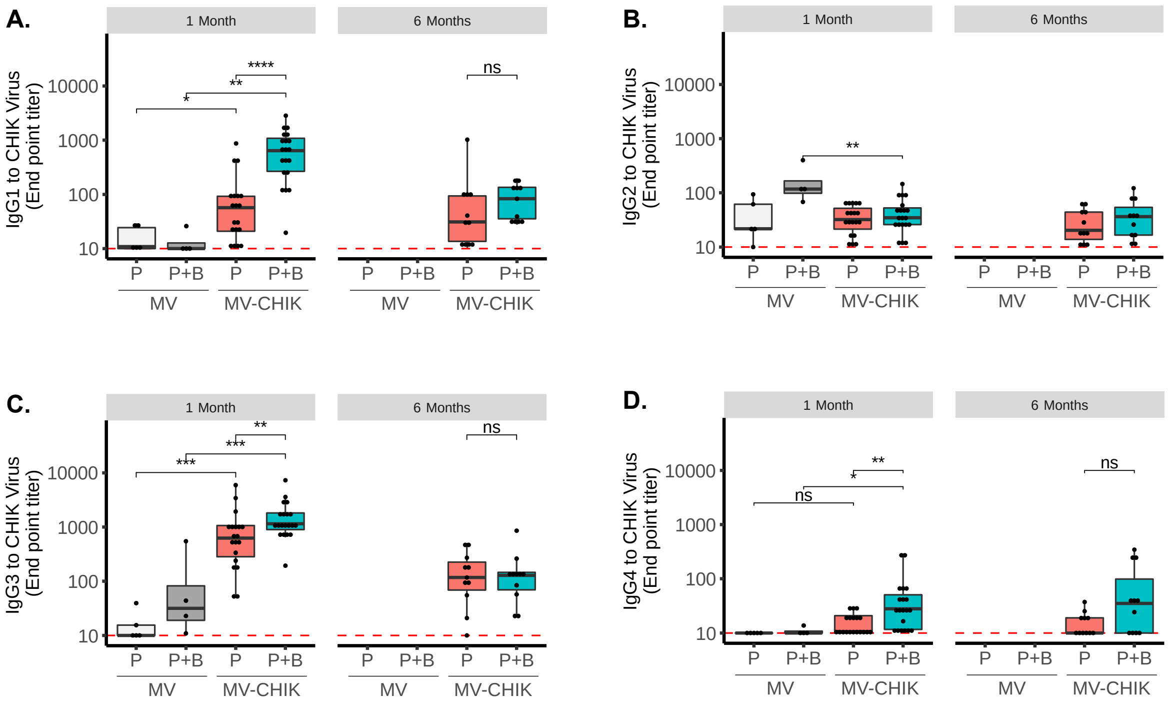

Supplementary Figure 2. CHIK virus-specific IgG subclass titres at 1- and 6-months post-vaccination with MV and MV-CHIK. A) IgG1, **B**) IgG2, **C**) IgG3 and **D**) IgG4 to CHIK virus following MV or MV-CHIK vaccination were measured as endpoint titres by ELISA. IgG subclass titres were compared between vaccinated groups using the Mann-Whitney *U* test.

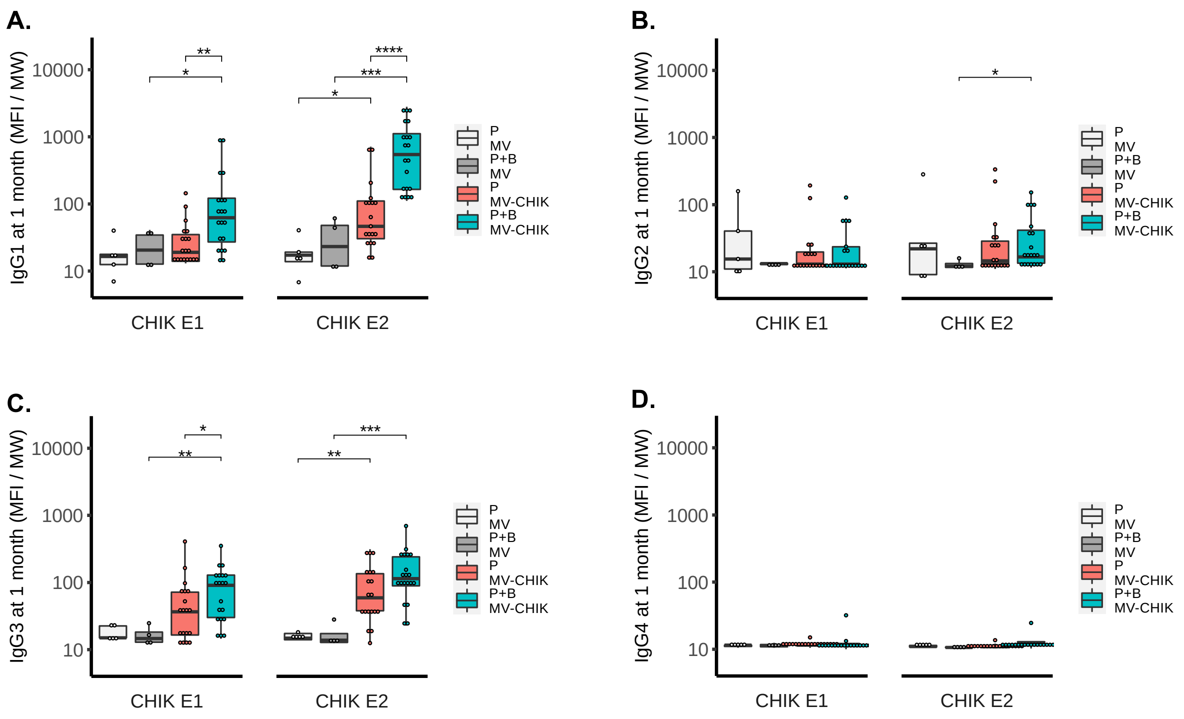

Supplementary Figure 3. CHIK E1 and E2-specific IgG subclass distribution post-vaccination with MV and MV-CHIK. A) IgG1, **B**) IgG2, **C**) IgG3 and **D**) IgG4 to CHIK E1 and E2 surface glycoproteins at 1-month post-vaccination with either MV or MV-CHIK (as assessed by Luminex immune-assay). IgG subclass (MFI) were compared between vaccinated groups using the Mann-Whitney *U* test.

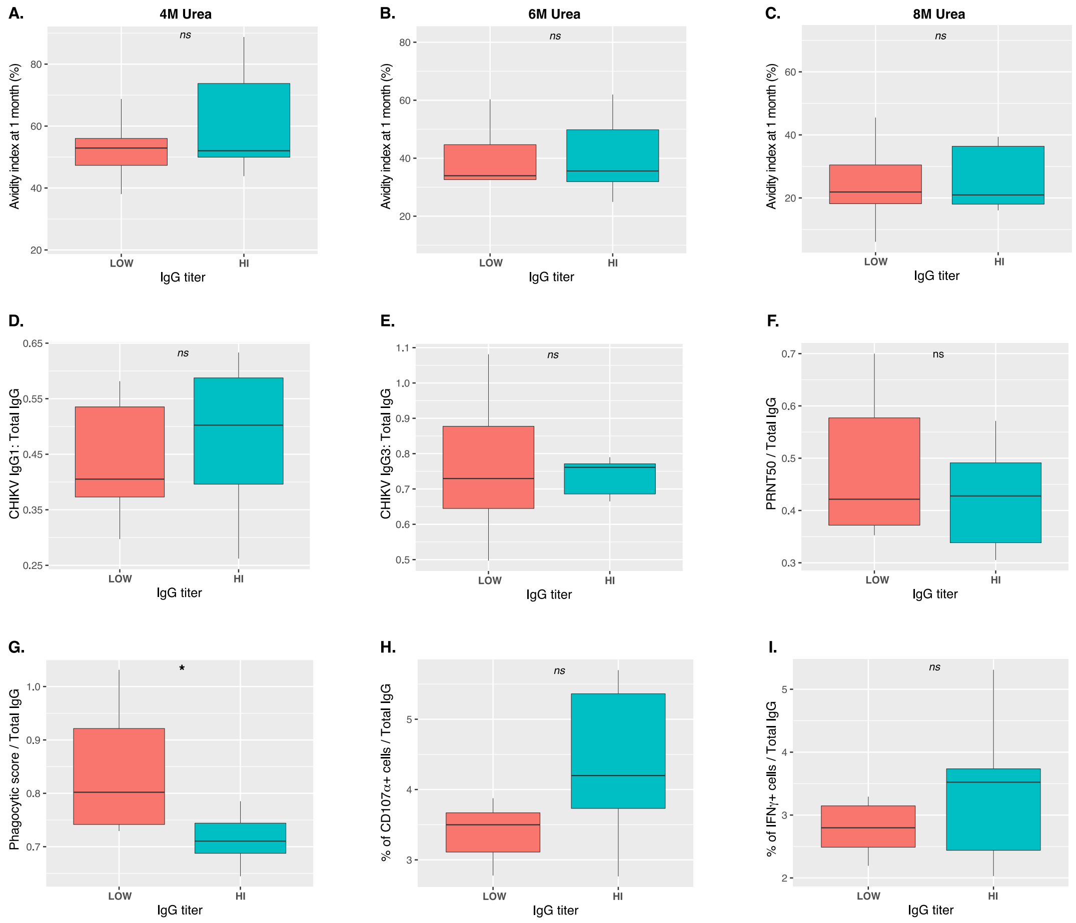

Supplementary Figure 4. Detailed comparison of antibody profiles at 1-month post-vaccination between vaccinees with low or high CHIKV-specific IgG titers. Antibody data for all vaccine serum samples (both P and P+B groups) were divided according to virus-specific IgG titers, i.e. low (less than median) and high (greater than median). The following antibody parameters were compared between low and high groups, avidity index with A) 4M, B) 6M and C) 8M urea, ratio of D) IgG1 and E) IgG3, F) virus neutralization (PRNT50 normalized to IgG), G) ADCP (phagocytic score normalized to IgG), H) ADNKA degranulation (% of CD107α+ cells normalized to IgG) and I) ADNKA cytokine release (% of IFNγ+ cells normalized to IgG).

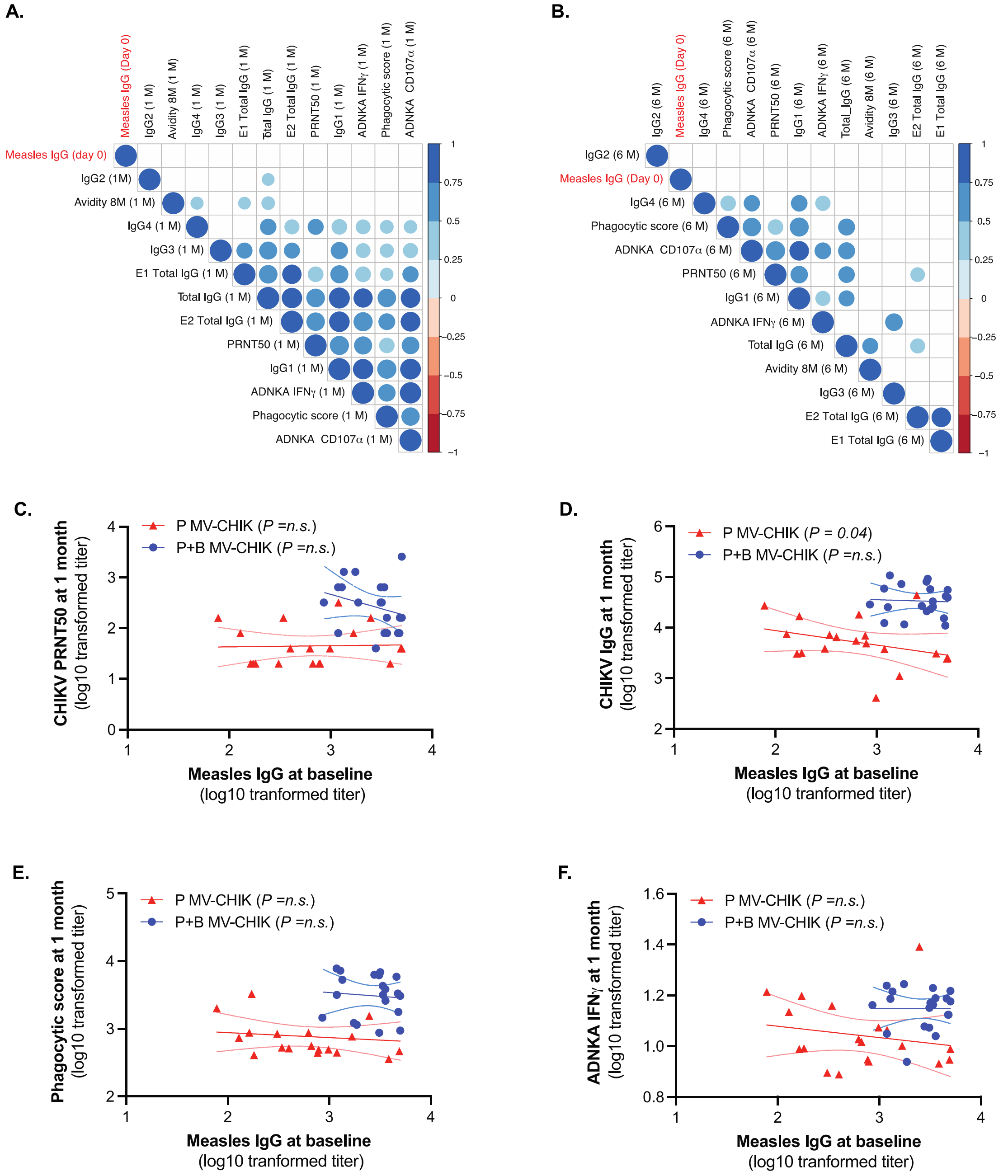

Supplementary Figure 5. Investigating the effects of baseline measles (MV)-specific antibody titres on CHIKV-specific responses in MV-CHIK vaccinated individuals. Correlation analysis with all study participants was conducted between MV-specific binding ELISA IgG at day 0 of study and several CHIKV-specific antibody parameters at 1 month (**A**) and 6 months (**B**). Only correlations with *P*-values>0.05 following Spearman correlation test is displayed in the above correlation matrices. **C-F**) Effects of MV-specific IgG at baseline (i.e. at pre-prime dose for and pre-boost dose for P MV-CHIK group and P+B MV-CHIK vaccinated groups, respectively) on CHIKV-specific antibody responses (i.e. CHIKV PRNT50 (**C**), CHIKV-IgG (**D**), ADCP as phagocytic score (**E**) and ADCC as ADNKA IFNγ (**F**)) in P MV-CHIK and P+B MV-CHIK vaccinated groups. *P*-values from Spearman correlation test on the non-log10 transformed data are displayed on the graphs.

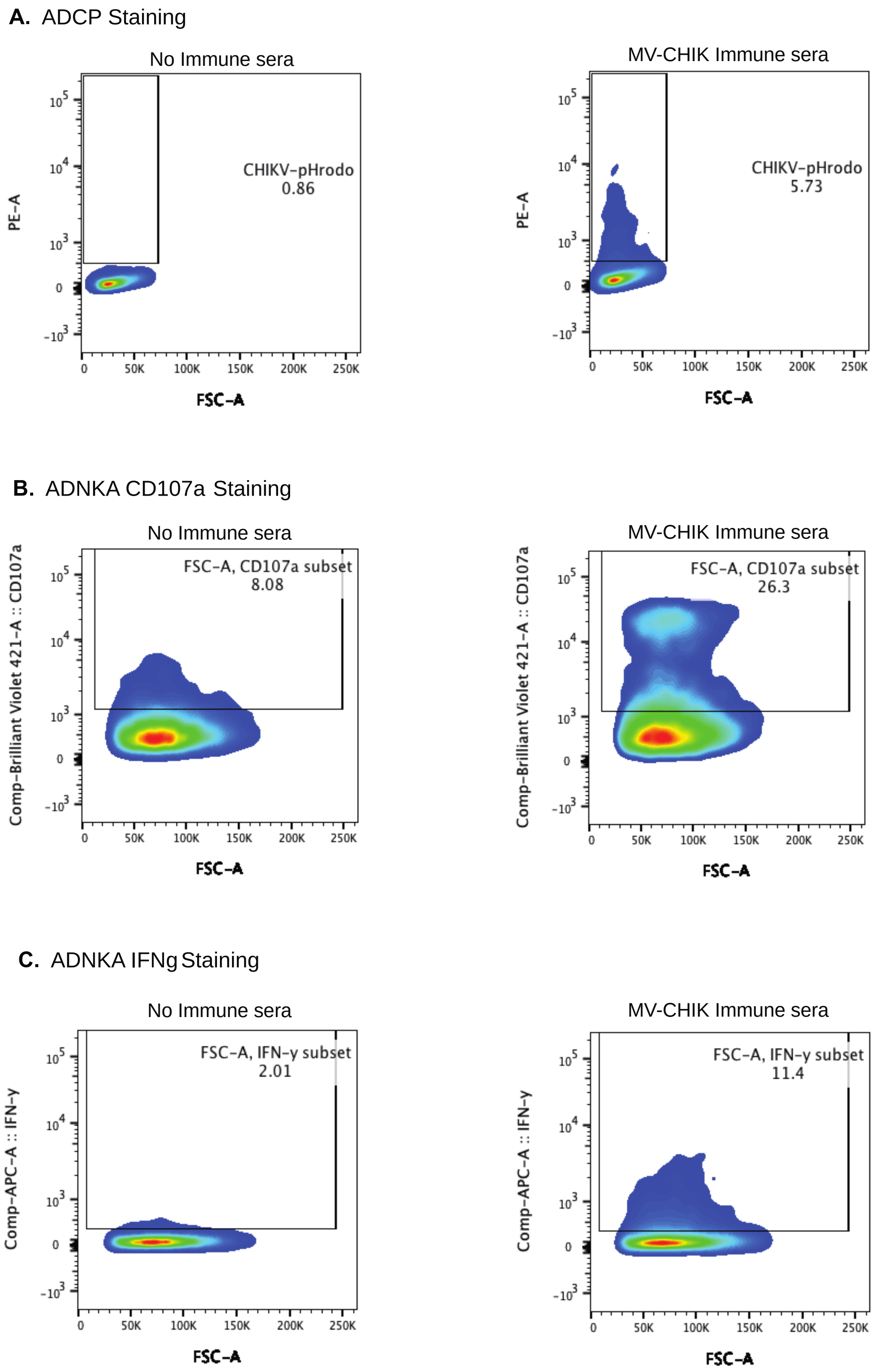

Supplementary Figure 6. Representative FACS staining from ADCP and ADNKA assays. Representative FACS staining with no immune sera and with MV-CHIK vaccinated sera are presented above for ADCP with pHrodo-labelled purified CHIKV virus (**A**), ADNKA CD107a (**B**) and ADNKA IFNg (**C**) against purified CHIKV virus.
